# Boyle, Glauber, and Newton: The Redintegration Experiment with Saltpeter

**DOI:** 10.1021/acsomega.4c00034

**Published:** 2024-03-27

**Authors:** Filip Adolf A. Buyse

**Affiliations:** Royal Flemish Chemical Society, Groenenborgerlaan, 171 2020 Antwerpen, Belgium

## Abstract

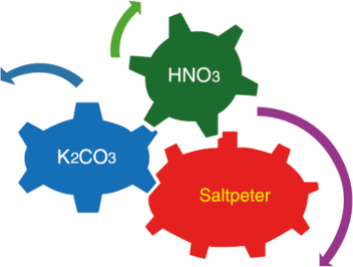

In 1661, Robert Boyle published his *Essay on Nitre*. In this famous essay, the author of *The Sceptical Chymist* (1661) introduces and promotes his new Corpuscular Philosophy. Central to this paper was the so-called redintegration or reconstitution experiment with saltpeter. However, this article shows that Boyle borrowed this experiment from Johann Rudolph Glauber, who had given it an alchemical interpretation. By contrast, opening the way to modern chemistry, Boyle gave it a new interpretation within the conceptual framework of his own Mechanical Philosophy. The redintegration experiment is not only important for the history of chemistry. It is very likely that the experiment also inspired Newton in his views on the composition of white light.

## 1 Robert Boyle’s Introduction of a New Philosophy

In one of the most seminal writings of his entire career, “The Father of Chemistry” introduces and defines for the very first time his “Corpuscular Philosophy”, a term he preferred as a synonym for Mechanical Philosophy:

That both parties agree in deducing all the Phaenomena of Nature from Matter and Local motion; I esteem‘d that notwithstanding these things wherein the Atomists and the Cartesians differed, they might be thought to agree in the main, and their Hypotheses might by a Person of a reconciling Disposition be looked’d on as, upon the matter, one Philosophy. Which because it explicates things by Corpuscles, or minute Bodies, may (not very unfitly) be call’d Corpuscular; though I sometimes style it the Phoenician Philosophy, because some ancient Writers inform us, that not only before *Epicurus* and *Democritus*, but ev’n before *Leucippus* taught in *Greece*, a Phoenician Naturalist [Moschus] was wont to give an account of the Phaenomena of Nature by the Motion and other Affections of the minute Particles of Matter. Which because they are obvious and very powerfull in Mechanical Engines, I sometimes also term it the Mechanical Hypothesis or Philosophy.^1^

In order to illustrate that his new philosophy was the right alternative for the scholastic philosophy based on the views of Aristotle and the alchemy based on Paracelsus, Robert Boyle (1627–1691) performed, in an essay of *Certain Physiological Essays* (1661) entitled *Essay On Nitre*, the so-called *redintegration* experiment with saltpeter (KNO_3_).^2^ With this experiment, Boyle aimed at demonstrating that substances like saltpeter could be decomposed and reconstituted like a pendulum clock and that all natural phenomena could be explained in terms of the mechanical properties of the corpuscles of bodies. However, Boyle’s use of this experiment raises several questions. What was the so-called *redintegration* experiment about? Did Boyle design this experiment himself, or was he rather inspired by somebody else? What was the context? Why did he use saltpeter for his experiment? What was innovative in Boyle’s interpretation of what happened? Is it possible to give a modern interpretation of the experiment? Is this experiment of historical importance? These are the kinds of questions that will be addressed in this paper.

## 2 The Redintegration Experiment with Saltpeter

In the first section of *On Nitre*, Boyle clarified that he wanted to perform an experiment with saltpeter because saltpeter was “one of the most Catholick of salts” and could be found in “Vegetable, Animal and even Mineral bodies”. Therefore, he believed that demonstrating his new philosophy with saltpeter would lead to “the discovery of the Nature of several other Bodies, and to the improvement of diverse parts of Natural Philosophy.”^2^ Saltpeter was “sold in Shops”, an important substance at the time, and was not only used for commercial and agricultural but also military purposes.

In the experiment, Boyle had placed a piece of glowing charcoal (primarily composed of carbon) in saltpeter. As a result, two substances were formed: volatile nitre, or Spirit of Nitre [*Spiritus Nitri*], and fix’d Nitre [*sal fixum*], which is “of an Alkalizate nature”. By then combining fixed nitre with Aqua fortis, “whose active part is little else than Spirit of Nitre”, in water, Boyle obtained saltpeter, the product with which he had started. These two processes can be summarized as follows:Step 1
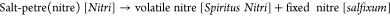
Step 2
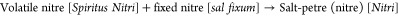
In a possible, more detailed modern reading, what the experimental phenomenon in question amounted to was an exothermic reaction of carbon (C) with saltpeter (KNO_3_) that gave form to several gases: carbon dioxide (CO_2_), nitrogen (N_2_), and nitrogen dioxide (NO_2_), which partially escaped from the vessel due to its high temperature. In addition to these gases, a white salt, in other words, *sal fixum* (K_2_CO_3_), was also formed in the original experiment. In the second step of the experiment, the spirit of nitre (NO_2_) was made to react with water (H_2_O) that was present in the vessel, and this gave form to nitric acid or *aqua fortis* (HNO_3_). Subsequently, nitric acid reacted with K_2_CO_3_ from the first reaction to form saltpeter, the substance with which Boyle had started.^3,4^ In a modern interpretation, the redintegration can be represented as follows:Step 1
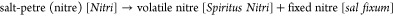

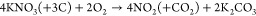
Step 2
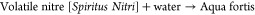

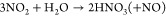
Step 3
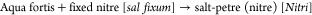

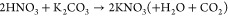
In addition, Boyle argued in section XII that he could, based on his new philosophy, explain all sensible qualities that accompanied the experiment in terms of the mechanical properties of the parts. This rather atomistic view was opposed to the traditional Peripatetic doctrine of qualities of bodies based on Aristotle’s ideas. According to this doctrine, bodies were conceived as a composite of substantial form and matter, and all qualities were regarded as real, intrinsic qualities of bodies. Importantly, in the following passage Boyle introduces the primary/secondary terminology of qualities of bodies in the English language, which states that secondary qualities of bodies had to be explained in terms of the spatial–temporal properties of its parts:

The reflections that may be made on this Experiment are more than I have either the skill or leisure to prosecute, and therefore I shall content myself to present you very succinctly with a few of those that do the most readily occur to my present thoughts. And first, this Experiment seems to afford us an instance by which we may discern that Motion, Figure, and Disposition of parts, and such like primary and mechanical Affections (if I may call so them) of Matter, may suffice to produce those more secondary Affections of Bodies which are wont to be called Sensible Qualities.^2^

Subsequently, in separate sections dedicated to each sense, he carefully demonstrates—in line with his definition—that all sensible qualities (secondary qualities) that accompanied the experiment are explainable in terms of shape, size, and motion of the minute parts that compose the bodies.^5^ The so-called primary/secondary distinction would be important in the work of most of the early modern philosophers. Moreover, according to Hume (1711–1776), it was the most important principle of modern philosophy.^6^

## 3 Scholastic Philosophy versus *Chymistry*

In 1661, Boyle’s secretary, Henri Oldenburg, sent the Latin translation of *On Nitre* to Spinoza. This was the start of an indirect correspondence between Boyle and the Dutch philosopher about the redintegration, highlighting Boyle’s intention to replace the peripatetic qualitative philosophy by his own corpuscular philosophy.^5^ Spinoza did not doubt that corpuscular philosophy was the correct alternative.^4,7^ Nevertheless, he gives a different corpuscular interpretation of the same experiment. In his view, saltpeter was not transformed into two different substances, which differed from saltpeter. Rather his interpretation of the redintegration experiment was what today we would call a physical process. More concretely, Spinoza defended the idea that the only difference between saltpeter (KNO_3_) and spirit of nitre (NO_2_/HNO_3_) is that the parts of saltpeter are at rest, whereas the parts of volatile nitre are in motion. Furthermore, as he argues, fixed nitre (*sal fixum*) is not a significant part of nitre but an impurity [*Foeces Nitri*]. In a modern reading, this seems to be quite correct given the fact that the niter refers to NO_*x*_ and *sal fixum* (K_2_CO_3_) does not contain NO_*x*_. Instead of being a compound that plays an active role in the experimental transformation, Spinoza insists that it is only an “*instrumentum*” [*tanquam instrumentum adhibetur*], which is comparable to what we know today as a catalyst (i.e., a compound that facilitates a reaction without directly participating in or being altered by it). In addition, Spinoza disagreed with Boyle’s experimental methodology. Furthermore, the rationalist argued that Boyle’s sophisticated experiment was completely redundant since Descartes and Bacon had already demonstrated Mechanical Philosophy in a convincing way. Further, he argued that daily experience shows sufficiently well the validity of the Mechanical Philosophy.^4^

When Spinoza’s discussion becomes, in Boyle’s view, too technical, Oldenburg intervenes on Boyle’s behalf and highlights that with his experiment Boyle only wanted to demonstrate that Corpuscular Philosophy was the right alternative for the qualitative natural philosophy based on substantial forms and real qualities:

But first he [Robert Boyle] wants you to know that it was not his intention to demonstrate that this was a truly philosophical and complete analysis of Nitre, bur rather to make the point that the common doctrine of substantial Forms and Qualities accepted in the Schools rests on a weak foundation, and that what they call the specific differences of things can be reduced to the magnitude, motion, rest and position of the parts.^7^

Traditionally, a body was conceived of as a compound of matter and substantial form. According to the Peripatetics, the latter was responsible for all qualities of bodies that were regarded to be real, intrinsic qualities that are sensed. By contrast, according to Boyle, in nature there are only corpuscles which only have mechanical properties (size, shape, and motion). The secondary qualities (including colors, savors, and odors) had to be explained in terms of these primary properties. In this context, Robert Boyle attacks numerous times the Peripatetics, not only in *Certain Physiological Essays* (1661) but also in *Forms and Qualities* (1666) and his *Enquiry* (1686).^8^

## 4 Between Chemistry and Alchemy

In the preface (which he had written just before publication of the volume) and in the last section (section 40) of *On nitre*, Boyle tries to convince his readers that he designed the redintegration himself and that he did not know the content of a newly published work by the German *chymist* Johann Rudolph R. Glauber (1604–1670).^2^ The Anglo-Irish natural philosopher is not exactly trustworthy, however, as he had already been inspired by the German *chymist* before. It is much more plausible that Boyle borrowed the experiment from Glauber. The German *chymist* had written before about the decomposition and recomposition of saltpeter.^9^ For instance, in the fourth part of his *Prosperitatis germaniæ* (1656–1661), published in Latin in 1659, two years before Boyle would publish *On Nitre*, he writes the following:

N.B. The acid Spirit of Niter does not dissolve sulfurous subjects, but mercurials onely: Contrarywise, the fix Niter doth not seize upon mercurial subjects but sulfurous ones; but the flame of Saltpeter performs both: which verily is wonderful, that things so unlike should in some few hours be extracted out of one and the same subject. For the corrosive Spirit prepared out of Salt-peter by Distillation, and likewise the fixed Salt, are most bitter enemies to each other, which ruinating and flaying one another, and being dead, return agen unto that which they were afore, and partakes of both natures; which the Ancient Philosophers do clearly point out unto us by the Griffon, which is headed and winged like an eagle, and the hinder part of its Body like a Lyon, as we have mentioned more at large in foregoing third part of the *Prosperity of Germany*.^10^

Obviously, the basic idea that saltpeter could be decomposed into two different substances (corrosive spirit and fixed salt), which differed from saltpeter, is already in Glauber’s text. Furthermore, Benjamin Worsley (1618–1673)—a member of the Hartlib Circle just like Boyle—had visited Glauber’s impressive lab in Amsterdam in 1648–1649 and had written a book about niter in the mid-1650s entitled *De nitro theses quaedam* wherein Glauber’s experiments with niter were mentioned.^9,11^ Importantly, in Worsley’s text we already read that “*It is certain that Salt-Peter hath Parts Volatill, inflammable and spirituous and parts fixed exceedingly causticke fiery and wonderfully detersive.*”^12^

However, Glauber’s explanation is very different from Boyle’s. Both *chymists* did not have the concept of a chemical reaction and a chemical element yet, so they had to articulate themselves in another way. This explains also that it would be anachronistic to make in this context a distinction between chemistry and alchemy. That is why this paper uses the terms *chymistry* and *chymists*.^17^

**Figure 1:**
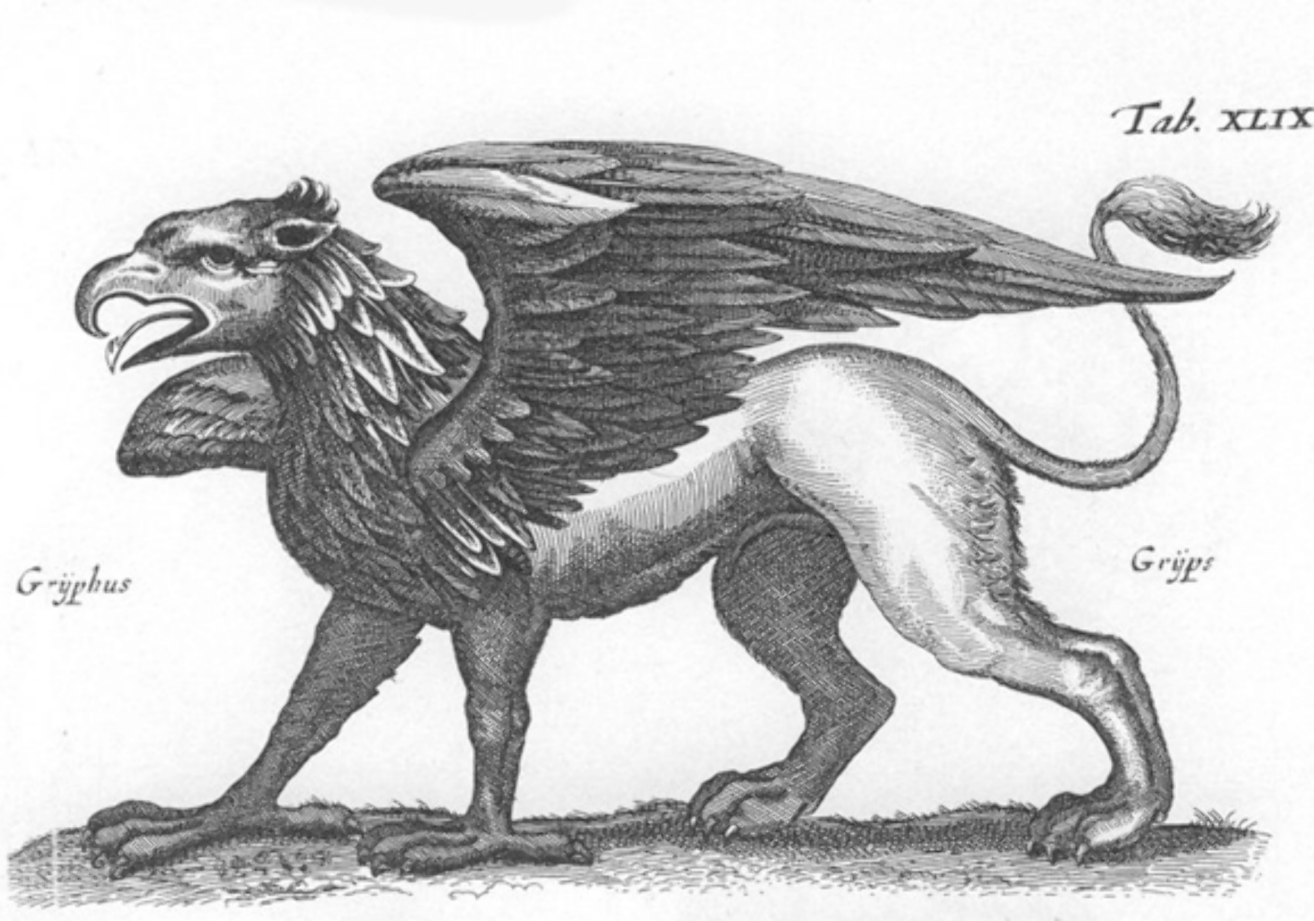
A picture of a Griffin, published in 1660 in Amsterdam in the first Dutch encyclopedia of animals.^13,14^

In a long passage at the end of part IV, Glauber compared niter (KNO_3_) with a mythological creature, the griffin (Figure 1), fixed salt (K_2_CO_3_) with a lion, and corrosive spirit (NO_2_/HNO_3_) with an eagle. So, in Glauber’s view, metaphorically, the transformation came down to a distillation of the eagle and the lion from the griffin and the subsequent reunion to the griffin.^10^ It is very important to note that Glauber uses alchemical terminology in his description, referring to the three primes (*the prima tria*) of Paracelsus’s alchemy: Mercury (volatile), Salt (solid), and Sulfur (flammable). Obviously, he conceived of saltpeter as a substance with a 3-fold nature and believed that through distillation he had analyzed it into its basic components: mercury, sulfur, and salt. By salt, alchemists designated every thing that was fixed in the fire. Furthermore, all inflammable substances were denominated by sulfur, and every substance that flies off without burning was denominated by mercury. Consequently, fixed salt (K_2_CO_3_) was linked with salt, niter (KNO_3_) with sulfur, and the corrosive spirit (NO_2_/HNO_3_) with mercury.

By contrast, Boyle explained the same transformation in a very different way. In line with his definition of Corpuscular Philosophy, he explains what happened in terms of the spatiotemporal properties of the corpuscles which compose all matter. He explained the redintegration rather by means of the metaphor of “a mechanical Engine”. In his view saltpeter was decomposed and recomposed just as a pendulum clock.^15^ The mechanical metaphor would also be very important in Boyle’s later work *Forms and Qualities* (1666), a prolongation of *On Nitre*.

Furthermore, Boyle correctly notes that Glauber had presented his experiment as a “bare Chymical Purification”, whereas Boyle speaks of his own attempt as a “Philosophical Redintegration”, indicating that for him this experiment was a way to illustrate, validate, and promote his new philosophy.^2^ Glauber, by contrast, applied redintegration in order to solve a traditional alchemical question. He defended the idea that he had found the universal dissolvent, the so-called alkahest:

[···] for my part, I remain constant in my Opinion, and say that saltpeter is a universal Dissolvent, and is able to dissolve all the things in the whole World, if it be made use of in three forms or shapes. Whatever the acid Spirit thereof, or the Eagle with its sharp Claws cannot effect, its fixed Salt, or the fiery Lyon will accomplish: and whatsoever is impossible to be done by these two, the Griffon which hath is rise from the Eagle and Lyon, will artificially perform.^10^

The quest for the alkahest was an extremely important question in 17th century alchemy. Saltpeter was a “fixed and caustic salt” that was flammable because it burned vividly with charcoal. Nitric acid was a powerful corrosive acid, and potassium carbonate was a saponifying base. Therefore, Glauber argued that a mixture of the three forms of niter could dissolve animal, vegetal, and mineral substances.

In addition, Glauber et al. had an economical agenda. In the *Prosperity of Germany* he wanted—after a long period of war—to show that saltpeter could be produced from waste that was abundantly available and could be subsequently transformed into all sorts of useful substances (including fertilizers, gunpowder, and pharmaceuticals), the production and sale of which would contribute to the prosperity of Germany.

## 5 Redintegration and Newton’s Optics

It is well-known that the young Newton (1643–1727) was influenced by Robert Boyle.^14,15^ However, it has only recently been argued that Boyle’s redintegration experiment played a significant role in Newton’s discovery that white light is a heterogeneous compound of immutable spectral colors. As William R. Newman put it: “it is little appreciated that Boyle’s analytical approach to *chymistry* had a profound impact on Newton’s optics in the second half of the 1660s, [···] Newton transferred Boyle’s analysis and resynthesis or “redintegration” of materials such as niter to the realm of light”.^16,18,19^

Newman’s thesis is based on several arguments.^18,19^ First of all, there is a structural similarity between Newton’s prism experiments and Boyle’s redintegration of saltpeter. In his experiments, Newton analyzed white light into its heterogeneous components and resynthesized it into white light, just as Boyle had decomposed saltpeter into its heterogeneous components and recomposed to saltpeter. Newton made his discovery around 1666, the year of the publication of Boyle’s *Forms and Qualities*.^19^ A document from Newton’s Laboratory Notebook written in this period, wherein Newton recorded the first experiments with the resynthesis of white light from spectral colors, contains extensive notes on Boyle’s *Forms and Qualities*, which appeared in the same period.^20^ Furthermore, the terminology that Newton employs in this context when describing this series of experiments in his optical lectures seems to give linguistic evidence of Boyle’s influence. For instance, in his *Lectiones opticae* as well as in the *Optica* Newton applies the Boylean term “*albedo redintegrata*” (redintegrated whiteness) when he explains that sunlight is reconstituted from spectral colors.^21^

Newton’s use of Boyle’s corpuscularian terminology indicates that colors are immutable, not further analyzable constituents of white light, just like the corpuscles featured in *chymical* experiments such as the redintegration experiment. This view was coherent with Newton’s corpuscular theory that proposes that light is a stream of a large number of particles known as corpuscles

## 6 Conclusions

Obviously, the redintegration experiment must be understood in the context of Boyle’s introduction of a new natural philosophy replacing the Paracelsian alchemy and the Peripatetic qualitative natural philosophy he had rejected. Boyle borrowed the experiment from Glauber, who gave the process an alchemical interpretation. By contrast, Boyle gave the experiment and its effects a new interpretation within the new conceptual framework of his corpuscular philosophy. In his new philosophy, Boyle was both directly and indirectly inspired by atomism and mechanics. Boyle’s interpretation very likely inspired Newton in his discovery and defense that white light was composed of immutable, spectral rays of colors.
